# A potential anti­cancer agent: 5-chloro-7-iodo-8-hy­droxy­quinolinium dichlorido(5-chloro-7-iodo­quinolin-8-olato-κ^2^ *N*,*O*)palladium(II) dihydrate

**DOI:** 10.1107/S1600536811040803

**Published:** 2011-10-08

**Authors:** Peter Vranec, Ivan Potočňák

**Affiliations:** a Department of Inorganic Chemistry, Faculty of Science, P.J. Šafárik University, Moyzesova 11, SK-041 54 Košice, Slovakia

## Abstract

The title Pd^II^ coordination compound, (C_9_H_6_ClINO)[PdCl_2_(C_9_H_4_ClINO)]·2H_2_O, was prepared as a potential anti­cancer agent. Its structure is ionic and consists of a square-planar [PdCl_2_(CQ)]^−^ complex anion (CQ is 5-chloro-7-iodo­quinolin-8-olate), with the Pd^II^ atom surrounded by two chloride ligands in a *cis* configuration and one *N*,*O*-bidentate CQ mol­ecule, a protonated anion of CQ as counter-cation and two non-coordinated water mol­ecules. The water mol­ecules are involved in O—H⋯O and N—H⋯O hydrogen bonds, which inter­connect the HCQ^+^ cations into a chain parallel to [010]. Apart from these inter­actions, the structure is also stabilized by face-to-face π–π inter­actions [centroid–centroid = 3.546 (3) Å], which occur between the phenolic parts of the complex anions and cations.

## Related literature

For background to square-planar complexes of platinum and palladium as potential chemotherapeutics, see: Bielawska *et al.* (2010 ▶); Bruijnincx & Sadler (2008 ▶); Ding *et al.* (2005 ▶); Garoufis *et al.* (2009 ▶). For structures of CQ complexes, see: Di Vaira *et al.* (2004 ▶) for [Cu(CQ)_2_] and [Zn(CQ)_2_(H_2_O)]·H_2_O·THF; Miyashita *et al.* (2005 ▶) for [ReCl_2_(CQ)O(PPh_3_)]. The structure of [Pd(8-HQ)_2_] (8-HQ = 8-hy­droxy­quinoline) was previously described by Prout & Wheeler (1966 ▶). For other related structures, see: Cui *et al.* (2009 ▶); Guney *et al.* (2011 ▶); Screnci & McKeage (1999 ▶); Yue *et al.* (2008 ▶); Kapteijn *et al.* (1996 ▶); Fazeli *et al.* (2009 ▶); Gniewek *et al.* (2006 ▶). Structures of complexes containing other halogen-derivatives of 8-HQ may also be found in the Cambridge Structural Database, see: Allen (2002 ▶). For π–π inter­actions, see: Janiak (2000 ▶).
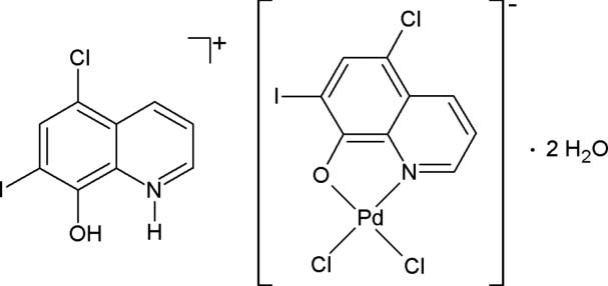


## Experimental

### 

#### Crystal data

(C_9_H_6_ClINO)[PdCl_2_(C_9_H_4_ClINO)]·2H_2_O
*M* *_r_* = 824.31
Monoclinic, 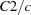

*a* = 34.3212 (10) Å
*b* = 7.7028 (2) Å
*c* = 18.4128 (5) Å
β = 104.455 (3)°
*V* = 4713.7 (2) Å^3^
*Z* = 8
Mo *K*α radiation
μ = 3.89 mm^−1^
*T* = 293 K
0.44 × 0.14 × 0.07 mm

#### Data collection

Oxford Diffraction Xcalibur Sapphire2 diffractometer
Absorption correction: analytical (*CrysAlis RED*; Oxford Diffraction, 2007 ▶) *T*_min_ = 0.555, *T*_max_ = 1.000
24287 measured reflections
4641 independent reflections
3888 reflections with *I* > 2σ(*I*)
*R*_int_ = 0.049

#### Refinement

*R*[*F* ^2^ > 2σ(*F* ^2^)] = 0.032
*wR*(*F* ^2^) = 0.080
*S* = 1.14
4641 reflections
287 parameters
H atoms treated by a mixture of independent and constrained refinement
Δρ_max_ = 0.61 e Å^−3^
Δρ_min_ = −0.87 e Å^−3^

### 

Data collection: *CrysAlis CCD* (Oxford Diffraction, 2007 ▶); cell refinement: *CrysAlis RED* (Oxford Diffraction, 2007 ▶); data reduction: *CrysAlis RED*; program(s) used to solve structure: *SHELXS97* (Sheldrick, 2008 ▶); program(s) used to refine structure: *SHELXL97* (Sheldrick, 2008 ▶) and *CALC-OH* (Nardelli, 1999 ▶); molecular graphics: *DIAMOND* (Brandenburg, 2001 ▶); software used to prepare material for publication: *SHELXL97*.

## Supplementary Material

Crystal structure: contains datablock(s) I, global. DOI: 10.1107/S1600536811040803/ff2030sup1.cif

Structure factors: contains datablock(s) I. DOI: 10.1107/S1600536811040803/ff2030Isup2.hkl

Additional supplementary materials:  crystallographic information; 3D view; checkCIF report

## Figures and Tables

**Table:** 

Pd1—N1	2.009 (4)
Pd1—O1	2.035 (3)
Pd1—Cl1	2.2711 (14)
Pd1—Cl2	2.3107 (14)

**Table:** 

N1—Pd1—O1	82.12 (15)
N1—Pd1—Cl1	94.01 (12)
O1—Pd1—Cl1	175.98 (10)
N1—Pd1—Cl2	175.90 (12)
O1—Pd1—Cl2	94.44 (10)
Cl1—Pd1—Cl2	89.47 (6)

**Table 2:** Hydrogen-bond geometry (Å, °)

*D*—H⋯*A*	*D*—H	H⋯*A*	*D*⋯*A*	*D*—H⋯*A*
O2—H2⋯O3	0.82	2.18	2.782 (7)	131
N2—H2*N*⋯O4*A*	0.82 (6)	1.92 (6)	2.737 (10)	174 (6)
N2—H2*N*⋯O4*B*	0.82 (6)	1.96 (6)	2.683 (9)	146 (6)
O4*A*—H1*O*4⋯O2	0.85	2.00	2.787 (10)	155
C28—H28⋯O3^i^	0.93	2.48	3.347 (8)	155

Symmetry code: (i) 
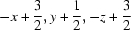
.

**Fig. 1.**
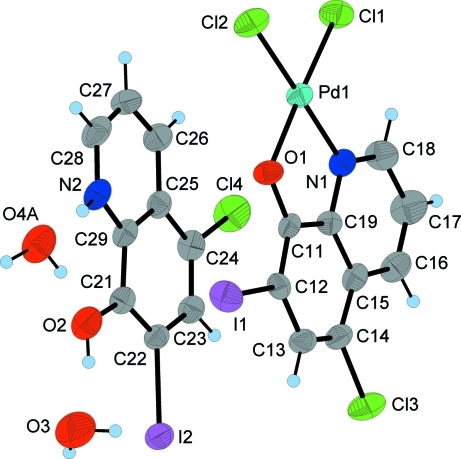
The structure of I. Displacement ellipsoids are drawn at the 50% probability for non-H atoms. H atoms are represented as small spheres of arbitrary radii. Only one position of the disordered O4 water molecule is shown.

**Fig. 2.**
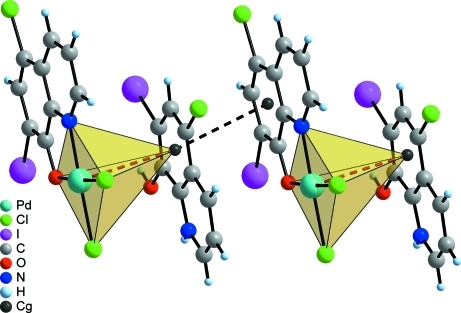
Parallel stacking of the cation and the complex anion enabling π-π interactions in I (shown by dashed lines); i = x, 1 + y, z. Possible penta-coordination of PdII is suggested.

**Fig. 3.**
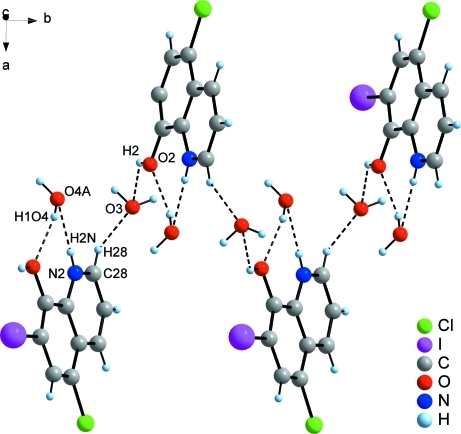
The system of hydrogen bonds (dashed lines) in I formed in the direction of b axis. Complex anions are not shown because of clarity.

## supplementary crystallographic information

### Comment

Square-planar complexes of platinum and palladium, as potential chemotherapeutics, are studied worldwide (Bruijnincx & Sadler, 2008 and Bielawska *et al.*, 2010). Unfortunately, many of these anticancer drugs exhibit significant side effects and their activity is relatively low (Screnci & McKeage, 1999). One of the approaches to overcome limitations connected with platinum- or palladium-based chemotherapy, new square-planar coordination compounds of these metals with biologically active ligands should be prepared. One of the examples of such ligand is 5-chloro-7-iodo-8-hydroxyquinoline (clioquinol, CQ), as it exhibits wide range of biological activity, including anticancer activity. CQ's favourable effect to human cancer cells is ascribed to its ability to chelate metal ions (Ding *et al.*, 2005). In our efforts to prepare novel square-planar complexes of Pt and Pd with clioquinol of Cat[MCl_2_(CQ)] (*M* = Pt or Pd; Cat = cation of +1 charge, such as Na^+^, K^+^ or Cs^+^) composition, we prepared crystals of HCQ[PdCl_2_(CQ)].2H_2_O (I) (HCQ = protonated molecule of CQ), which we believe has an increased anticancer activity. Here we present the structure of the title compound.

The molecular structure of the ionic HCQ[PdCl_2_(CQ)].2H_2_O (I) compound consists of discrete [PdCl_2_(CQ)]^-^ anion in which the central Pd^II^ atom has a distorted square-planar configuration, protonated molecule of CQ (HCQ^+^) as cation, and two non-coordinated water molecules (Fig. 1). Complex anion is formed by Pd^II^ atom which is surrounded by two chlorido ligands in *cis*- configuration at 2.271 (1) (Pd1—Cl1) and 2.311 (1) Å (Pd1—Cl2) distances, which are close to Pd—Cl distances observed in other square planar Pd^II^ complexes (Cui *et al.*, 2009), and one bidentately coordinated CQ molecule. This is bound to Pd^II^ atom by nitrogen atom of pyridine part and oxygen atom, which is ready to coordinate after deprotonation of the CQ's hydroxyl group in phenolic part; the Pd1—N1 (2.009 (4) Å) and Pd1—O1 (2.035 (3) Å) distances are normal (Yue *et al.*, 2008). Both the coordinated and free protonated CQ molecules are nearly planar, with the largest deviation of atoms from the mean planes through the aromatic rings being 0.05 (1) Å. The geometric parameters within the individual rings resemble those found in similar compounds containing pyridine and phenolic rings (Guney *et al.*, 2011 and Kapteijn *et al.*, 1996). The C—*X* bonds (*X* = Cl and I; 1.742 (10) and 2.098 (2) Å in average, respectively) are usual for single C_sp2_—*X* bonds (Fazeli *et al.*, 2009 and Gniewek *et al.*, 2006).

Besides the ionic forces, the structure is also stabilized by π-π interactions and hydrogen bonds. π-π interactions occur between the phenolic parts of the complex anion and the cation. The distance between centroids of these parts (*Cg*_An_—*Cg*_Cat_ = 3.546 (3) Å) and angle between normal to the plane and vector connecting the two centroids (16.46°) are consistent with the values typical for the face-to-face π–π interactions (Janiak, 2000). Moreover, the distance between Pd1 atom and *Cg*_Cat_^i^ of another adjacent cation (i = *x*, 1 + *y*, *z*) of 3.497 Å and the angle between normal to the plane of HCQ^+^ cation and vector connecting *Cg*_Cat_^i^ and Pd1 of 171.96° indicate possible η^6^ semi coordination of the phenyl ring of the cation. Thus the coordination number of Pd atom can be considered as 4 + 1 with a tetragonal pyramidal coordination polyhedron. Due to these intermolecular contacts the cations and anions are linked to form a chain parallel with [010] (Fig. 2).

Two uncoordinated water molecules interconnect the HCQ^+^ cations *via* hydrogen bonds into a chain running along [010] (Fig. 3). Distances and angles characterizing these bonds are summarized in Table 2.

### Experimental

Ethanolic solution of PdCl_2_ prepared from 0.2 cm^3^ 40% water solution of PdCl_2_ in 8 cm^3^ of ethanol (0.048 g PdCl_2_; 0.27 mmol) was cooled down to -15 °C and mixed with a cold (-5 °C) THF solution of CQ (0.17 g CQ dissolved in 15 cm^3^ of THF; 0.54 mmol). Resulting solution was stirred at -15 °C for a while and then a cold (3 °C) aqueous solution of CsCl (0.046 g of CsCl dissolved in 2 cm^3^ of water; 0.27 mmol) was added. Yellow precipitation of I, which formed immediately after mixing, was filtered off, dried on air and analyzed by IR and elemental analysis. Mother liquor was left for crystallization in refrigerator at -5 °C and after few days we obtained a small amount of orange-red crystals of I. Crystals were filtered off, dried on air and analyzed by IR spectroscopy to prove their identity with the precipitation.

### Refinement

H atoms of the CQ moieties were inserted in calculated positions appropriate for the data collection temperature with isotropic displacement parameters riding on that of the parent C and O atoms, *U*_iso_(H) = 1.2Ueq(C) and *U*_iso_(H) = 1.5Ueq(O). The hydrogen atom coordinated on the N2 atom in HCQ^+^ was found in the difference electron map and refined freely, water H atoms were found with the program *CALC*-OH (Nardelli, 1999) and were refined with fixed bond distances and angles. Hydrogen atoms could be found only for one disordered position (O4A).

### Crystal data

**Table:**

(C_9_H_6_ClINO)[PdCl_2_(C_9_H_4_ClINO)]·2H_2_O	*F*(000) = 3104
*M**_r_* = 824.31	*D*_x_ = 2.323 Mg m^−^^3^
Monoclinic, *C*2/*c*	Mo *K*α radiation, λ = 0.71073 Å
Hall symbol: -C 2yc	Cell parameters from 14809 reflections
*a* = 34.3212 (10) Å	θ = 3.0–29.6°
*b* = 7.7028 (2) Å	µ = 3.89 mm^−^^1^
*c* = 18.4128 (5) Å	*T* = 293 K
β = 104.455 (3)°	Needle, orange-red
*V* = 4713.7 (2) Å^3^	0.44 × 0.14 × 0.07 mm
*Z* = 8	

### Data collection

**Table:**

Oxford Diffraction Xcalibur Sapphire2 diffractometer	4641 independent reflections
Radiation source: fine-focus sealed tube	3888 reflections with *I* > 2σ(*I*)
graphite	*R*_int_ = 0.049
Detector resolution: 8.3438 pixels mm^-1^	θ_max_ = 26.0°, θ_min_ = 3.0°
ω scans	*h* = −42→42
Absorption correction: analytical (*CrysAlis RED*; Oxford Diffraction, 2007)	*k* = −9→9
*T*_min_ = 0.555, *T*_max_ = 1.000	*l* = −22→22
24287 measured reflections	

### Refinement

**Table:**

Refinement on *F*^2^	Primary atom site location: structure-invariant direct methods
Least-squares matrix: full	Secondary atom site location: difference Fourier map
*R*[*F*^2^ > 2σ(*F*^2^)] = 0.032	Hydrogen site location: inferred from neighbouring sites
*wR*(*F*^2^) = 0.080	H atoms treated by a mixture of independent and constrained refinement
*S* = 1.14	*w* = 1/[σ^2^(*F*_o_^2^) + (0.0265*P*)^2^ + 28.2625*P*] where *P* = (*F*_o_^2^ + 2*F*_c_^2^)/3
4641 reflections	(Δ/σ)_max_ = 0.002
287 parameters	Δρ_max_ = 0.61 e Å^−^^3^
0 restraints	Δρ_min_ = −0.87 e Å^−^^3^

### Special details

**Table:**

Experimental. CrysAlis RED, Oxford Diffraction (2007), Analytical numeric absorption correction using a multifaceted crystal model.
Geometry. All e.s.d.'s (except the e.s.d. in the dihedral angle between two l.s. planes) are estimated using the full covariance matrix. The cell e.s.d.'s are taken into account individually in the estimation of e.s.d.'s in distances, angles and torsion angles; correlations between e.s.d.'s in cell parameters are only used when they are defined by crystal symmetry. An approximate (isotropic) treatment of cell e.s.d.'s is used for estimating e.s.d.'s involving l.s. planes.
Refinement. Refinement of *F*^2^ against ALL reflections. The weighted *R*-factor *wR* and goodness of fit *S* are based on *F*^2^, conventional *R*-factors *R* are based on *F*, with *F* set to zero for negative *F*^2^. The threshold expression of *F*^2^ > σ(*F*^2^) is used only for calculating *R*-factors(gt) *etc*. and is not relevant to the choice of reflections for refinement. *R*-factors based on *F*^2^ are statistically about twice as large as those based on *F*, and *R*- factors based on ALL data will be even larger.

### Fractional atomic coordinates and isotropic or equivalent isotropic displacement parameters (Å^2^)

**Table:**

	*x*	*y*	*z*	*U*_iso_*/*U*_eq_	Occ. (<1)
I1	0.699063 (11)	0.67835 (5)	0.58026 (2)	0.04530 (12)	
Pd1	0.627824 (11)	0.95775 (5)	0.77655 (2)	0.02970 (11)	
Cl1	0.59303 (5)	1.0787 (2)	0.85369 (8)	0.0471 (4)	
Cl2	0.68728 (4)	1.0204 (2)	0.86375 (8)	0.0483 (4)	
I2	0.611094 (11)	0.16194 (5)	0.507511 (19)	0.03868 (11)	
Cl3	0.53299 (5)	0.6203 (2)	0.43378 (8)	0.0515 (4)	
Cl4	0.54308 (4)	0.4700 (3)	0.72671 (9)	0.0574 (4)	
O1	0.65529 (10)	0.8421 (5)	0.70322 (19)	0.0343 (8)	
O2	0.69038 (11)	0.2391 (6)	0.6466 (2)	0.0469 (10)	
H2	0.6854	0.1925	0.6052	0.070*	
N1	0.57824 (12)	0.9057 (6)	0.6950 (2)	0.0329 (10)	
C13	0.60872 (16)	0.6631 (7)	0.5168 (3)	0.0345 (12)	
H3	0.6161	0.6098	0.4769	0.041*	
N2	0.69550 (14)	0.4180 (6)	0.7764 (3)	0.0373 (11)	
H2N	0.7156 (18)	0.398 (8)	0.761 (3)	0.045*	
C12	0.63853 (15)	0.7155 (6)	0.5801 (3)	0.0288 (10)	
C14	0.56918 (16)	0.6898 (7)	0.5132 (3)	0.0346 (12)	
C11	0.62914 (14)	0.7916 (6)	0.6417 (3)	0.0274 (10)	
C28	0.70063 (18)	0.4935 (8)	0.8424 (3)	0.0459 (15)	
H28	0.7265	0.5133	0.8719	0.055*	
C29	0.65883 (15)	0.3807 (7)	0.7305 (3)	0.0310 (11)	
C19	0.58701 (14)	0.8219 (6)	0.6352 (3)	0.0279 (10)	
C25	0.62410 (15)	0.4374 (7)	0.7528 (3)	0.0315 (11)	
C23	0.58380 (15)	0.3332 (7)	0.6341 (3)	0.0331 (11)	
H23	0.5587	0.3198	0.6008	0.040*	
C18	0.54040 (16)	0.9481 (8)	0.6920 (3)	0.0441 (14)	
H8	0.5346	1.0081	0.7319	0.053*	
C22	0.61854 (15)	0.2761 (6)	0.6136 (3)	0.0302 (11)	
C24	0.58658 (15)	0.4075 (7)	0.7019 (3)	0.0337 (11)	
C21	0.65593 (15)	0.2950 (7)	0.6611 (3)	0.0304 (11)	
C16	0.51643 (16)	0.8190 (8)	0.5717 (3)	0.0418 (13)	
H6	0.4953	0.7889	0.5311	0.050*	
C15	0.55647 (15)	0.7739 (7)	0.5712 (3)	0.0328 (11)	
C27	0.66770 (19)	0.5433 (8)	0.8681 (3)	0.0451 (14)	
H27	0.6713	0.5941	0.9151	0.054*	
C26	0.62989 (17)	0.5169 (7)	0.8236 (3)	0.0388 (13)	
H26	0.6077	0.5519	0.8403	0.047*	
C17	0.50877 (17)	0.9044 (9)	0.6298 (3)	0.0538 (16)	
H7	0.4825	0.9349	0.6292	0.065*	
O4A	0.7649 (2)	0.3424 (13)	0.7353 (5)	0.0556 (18)	0.50
H1O4	0.7444	0.3321	0.6986	0.083*	0.50
H2O4	0.7819	0.2658	0.7303	0.083*	0.50
O4B	0.7706 (2)	0.4711 (14)	0.7626 (5)	0.0556 (18)	0.50
O3	0.72251 (16)	0.1567 (7)	0.5263 (3)	0.0780 (16)	
H2O3	0.7047	0.0789	0.5129	0.117*	
H1O3	0.7125	0.2474	0.5028	0.117*	

### Atomic displacement parameters (Å^2^)

**Table:**

	*U*^11^	*U*^22^	*U*^33^	*U*^12^	*U*^13^	*U*^23^
I1	0.0356 (2)	0.0526 (2)	0.0522 (2)	0.00153 (17)	0.01937 (17)	−0.00573 (18)
Pd1	0.0312 (2)	0.0324 (2)	0.02578 (18)	−0.00258 (17)	0.00749 (15)	0.00048 (16)
Cl1	0.0538 (9)	0.0550 (9)	0.0381 (7)	0.0005 (7)	0.0218 (7)	−0.0047 (6)
Cl2	0.0436 (8)	0.0610 (9)	0.0350 (7)	−0.0094 (7)	−0.0002 (6)	−0.0030 (7)
I2	0.0445 (2)	0.0382 (2)	0.03188 (18)	−0.00183 (16)	0.00673 (15)	0.00035 (15)
Cl3	0.0471 (9)	0.0655 (10)	0.0352 (7)	−0.0147 (7)	−0.0020 (6)	−0.0088 (7)
Cl4	0.0315 (7)	0.0886 (12)	0.0561 (9)	0.0108 (8)	0.0184 (7)	0.0014 (9)
O1	0.0263 (18)	0.045 (2)	0.0298 (18)	−0.0024 (16)	0.0042 (15)	−0.0069 (16)
O2	0.036 (2)	0.066 (3)	0.041 (2)	0.012 (2)	0.0132 (18)	0.006 (2)
N1	0.028 (2)	0.039 (2)	0.032 (2)	−0.0007 (19)	0.0082 (18)	0.0029 (19)
C13	0.045 (3)	0.033 (3)	0.026 (2)	−0.003 (2)	0.010 (2)	0.001 (2)
N2	0.028 (2)	0.044 (3)	0.036 (2)	−0.004 (2)	−0.0001 (19)	0.008 (2)
C12	0.028 (3)	0.027 (3)	0.034 (2)	0.002 (2)	0.011 (2)	0.001 (2)
C14	0.039 (3)	0.034 (3)	0.026 (2)	−0.006 (2)	0.000 (2)	0.001 (2)
C11	0.028 (3)	0.028 (3)	0.025 (2)	−0.001 (2)	0.003 (2)	0.006 (2)
C28	0.043 (3)	0.044 (3)	0.040 (3)	−0.012 (3)	−0.011 (3)	0.006 (3)
C29	0.027 (3)	0.033 (3)	0.031 (3)	−0.006 (2)	0.001 (2)	0.007 (2)
C19	0.028 (3)	0.028 (3)	0.028 (2)	−0.003 (2)	0.008 (2)	−0.001 (2)
C25	0.035 (3)	0.029 (3)	0.030 (2)	−0.002 (2)	0.008 (2)	0.007 (2)
C23	0.026 (3)	0.036 (3)	0.034 (3)	−0.003 (2)	0.003 (2)	0.002 (2)
C18	0.033 (3)	0.058 (4)	0.044 (3)	0.004 (3)	0.015 (3)	−0.006 (3)
C22	0.034 (3)	0.030 (3)	0.026 (2)	−0.002 (2)	0.005 (2)	0.002 (2)
C24	0.024 (2)	0.040 (3)	0.039 (3)	−0.002 (2)	0.013 (2)	0.007 (2)
C21	0.028 (3)	0.035 (3)	0.030 (2)	−0.001 (2)	0.010 (2)	0.005 (2)
C16	0.028 (3)	0.054 (4)	0.039 (3)	−0.003 (3)	0.000 (2)	0.001 (3)
C15	0.030 (3)	0.036 (3)	0.031 (3)	−0.003 (2)	0.005 (2)	0.007 (2)
C27	0.059 (4)	0.045 (3)	0.028 (3)	0.000 (3)	0.005 (3)	−0.004 (2)
C26	0.044 (3)	0.042 (3)	0.032 (3)	0.000 (3)	0.013 (2)	0.005 (2)
C17	0.027 (3)	0.079 (5)	0.054 (4)	0.001 (3)	0.008 (3)	−0.004 (3)
O4A	0.030 (3)	0.082 (6)	0.055 (4)	0.009 (4)	0.010 (3)	0.010 (4)
O4B	0.030 (3)	0.082 (6)	0.055 (4)	0.009 (4)	0.010 (3)	0.010 (4)
O3	0.083 (4)	0.075 (4)	0.063 (3)	−0.007 (3)	−0.007 (3)	−0.007 (3)

### Geometric parameters (Å, °)

**Table:**

I1—C12	2.096 (5)	C28—H28	0.9300
Pd1—N1	2.009 (4)	C29—C21	1.420 (7)
Pd1—O1	2.035 (3)	C29—C25	1.423 (7)
Pd1—Cl1	2.2711 (14)	C19—C15	1.416 (7)
Pd1—Cl2	2.3107 (14)	C25—C26	1.409 (7)
I2—C22	2.099 (5)	C25—C24	1.410 (7)
Cl3—C14	1.749 (5)	C23—C24	1.354 (7)
Cl4—C24	1.735 (5)	C23—C22	1.408 (7)
O1—C11	1.316 (6)	C23—H23	0.9300
O2—C21	1.347 (6)	C18—C17	1.407 (8)
O2—H2	0.8200	C18—H8	0.9300
N1—C18	1.327 (7)	C22—C21	1.369 (7)
N1—C19	1.373 (6)	C16—C17	1.338 (8)
C13—C14	1.358 (8)	C16—C15	1.419 (7)
C13—C12	1.405 (7)	C16—H6	0.9300
C13—H3	0.9300	C27—C26	1.366 (8)
N2—C28	1.319 (7)	C27—H27	0.9300
N2—C29	1.360 (6)	C26—H26	0.9300
N2—H2N	0.82 (6)	C17—H7	0.9300
C12—C11	1.385 (7)	O4A—H1O4	0.8502
C14—C15	1.409 (7)	O4A—H2O4	0.8499
C11—C19	1.440 (7)	O3—H2O3	0.8488
C28—C27	1.384 (9)	O3—H1O3	0.8483
			
N1—Pd1—O1	82.12 (15)	C26—C25—C24	125.5 (5)
N1—Pd1—Cl1	94.01 (12)	C26—C25—C29	117.7 (5)
O1—Pd1—Cl1	175.98 (10)	C24—C25—C29	116.7 (5)
N1—Pd1—Cl2	175.90 (12)	C24—C23—C22	120.6 (5)
O1—Pd1—Cl2	94.44 (10)	C24—C23—H23	119.7
Cl1—Pd1—Cl2	89.47 (6)	C22—C23—H23	119.7
C11—O1—Pd1	111.8 (3)	N1—C18—C17	121.5 (5)
C21—O2—H2	109.5	N1—C18—H8	119.2
C18—N1—C19	119.4 (4)	C17—C18—H8	119.2
C18—N1—Pd1	128.3 (4)	C21—C22—C23	121.1 (5)
C19—N1—Pd1	112.3 (3)	C21—C22—I2	121.1 (4)
C14—C13—C12	120.6 (5)	C23—C22—I2	117.8 (4)
C14—C13—H3	119.7	C23—C24—C25	121.6 (5)
C12—C13—H3	119.7	C23—C24—Cl4	119.5 (4)
C28—N2—C29	123.7 (5)	C25—C24—Cl4	118.8 (4)
C28—N2—H2N	118 (4)	O2—C21—C22	124.6 (5)
C29—N2—H2N	118 (4)	O2—C21—C29	117.4 (4)
C11—C12—C13	122.1 (5)	C22—C21—C29	118.0 (5)
C11—C12—I1	119.3 (4)	C17—C16—C15	120.6 (5)
C13—C12—I1	118.6 (4)	C17—C16—H6	119.7
C13—C14—C15	121.8 (5)	C15—C16—H6	119.7
C13—C14—Cl3	119.1 (4)	C14—C15—C19	116.5 (5)
C15—C14—Cl3	119.0 (4)	C14—C15—C16	126.9 (5)
O1—C11—C12	125.6 (4)	C19—C15—C16	116.6 (5)
O1—C11—C19	118.6 (4)	C26—C27—C28	119.3 (5)
C12—C11—C19	115.7 (4)	C26—C27—H27	120.4
N2—C28—C27	120.3 (5)	C28—C27—H27	120.4
N2—C28—H28	119.9	C27—C26—C25	120.9 (5)
C27—C28—H28	119.9	C27—C26—H26	119.6
N2—C29—C21	120.2 (5)	C25—C26—H26	119.6
N2—C29—C25	117.9 (5)	C16—C17—C18	120.2 (5)
C21—C29—C25	121.8 (4)	C16—C17—H7	119.9
N1—C19—C15	121.7 (4)	C18—C17—H7	119.9
N1—C19—C11	115.2 (4)	H1O4—O4A—H2O4	107.7
C15—C19—C11	123.1 (4)	H2O3—O3—H1O3	105.2

### Hydrogen-bond geometry (Å, °)

**Table:**

*D*—H···*A*	*D*—H	H···*A*	*D*···*A*	*D*—H···*A*
O2—H2···O3	0.82	2.18	2.782 (7)	131.
N2—H2N···O4A	0.82 (6)	1.92 (6)	2.737 (10)	174 (6)
N2—H2N···O4B	0.82 (6)	1.96 (6)	2.683 (9)	146 (6)
O4A—H1O4···O2	0.85	2.00	2.787 (10)	155.
C28—H28···O3^i^	0.93	2.48	3.347 (8)	155.

Symmetry codes: (i) −*x*+3/2, *y*+1/2, −*z*+3/2.
